# 

**Published:** 1898-06

**Authors:** 


### BOOKS RECEIVED.

Accident and Injury. Their Relations to Diseases of the Nervous System. By Pearce Bailey, A. M., M. D., Attending Physician to the Department of Correction and to the Almshouse and Incurable Hospitals, New York, etc., etc. Octavo, pp. xii.—430. New York: D. Appleton & Co. 1898.

Public Health Reports (formerly Abstract of Sanitary Reports). Issued by the Supervising Surgeon-General, Marine Hospital Service. Volume XII., Nos. 1–53. Washington: Government Printing Office. 1898.

A Manual of Hygiene and Sanitation. By Seneca Egbert, A. M., M. D., Professor of Hygiene in the Medico-Chirurgical College of Philadelphia. In one 12mo volume of 360 pp. with sixty-three engravings. Cloth, $2.25, net. Philadelphia and New York: Lea Brothers & Co. 1898.

Report of the Health Department of the City and County of San Francisco for the fiscal year ending June 30, 1897. Edmond Godchaux, Secretary, San Francisco. 1897.

A Manual of Instruction in the Principles of Prompt Aid to the Injured. Including a chapter on hygiene and the drill regulations for the hospital corps, U. S. A. By Alvah H. Doty, M. D., Health Officer of the Port of New York, etc. Duodecimo, pp. xvi.—302. New York: D. Appleton & Co. 1898.

Brief Essays on Orthopedic Surgery. By Newton M. Shaeffer, M. D., Surgeon-in-chief to the New York Orthopedic Dispensary and Hospital, etc. Duodecimo, pp. 81. New York: D. Appleton & Co. 1898.

Transactions of the Southern Surgical and Gynecological Society. Tenth Annual Meeting, held at St. Louis, Mo., November 9, 10 and 11, 1897. William E. B. Davis, M. D., Secretary. Published by the Association. 1898.

